# Titanium dioxide nanoparticles enhance the detrimental effect of polystyrene nanoplastics on cell and plant physiology of *Vicia lens* (L.) Coss. & Germ. seedlings

**DOI:** 10.3389/fpls.2024.1391751

**Published:** 2024-05-28

**Authors:** Carmelina Spanò, Lucia Giorgetti, Stefania Bottega, Simonetta Muccifora, Monica Ruffini Castiglione

**Affiliations:** ^1^ Department of Biology, University of Pisa, Pisa, Italy; ^2^ Centre for Climate Change Impact, University of Pisa, Pisa, Italy; ^3^ Institute of Agricultural Biology and Biotechnology, National Research Council, Pisa, Italy; ^4^ Department of Life Sciences, University of Siena, Siena, Italy

**Keywords:** fluorescent polystyrene nanoplastics, titanium dioxide nanoparticles, co-contamination, lentil, seedling growth, cyto/genotoxicity, oxidative stress, histochemistry

## Abstract

Polystyrene nanoplastics and titanium dioxide nanoparticles are widely spread in all environments, often coexisting within identical frameworks. Both these contaminants can induce negative effects on cell and plant physiology, giving concerns on their possible interaction which could increase each other’s harmful effects on plants. Despite the urgency of this issue, there is very little literature addressing it. To evaluate the potential risk of this co-contamination, lentil seeds were treated for five days with polystyrene nanoplastics and titanium dioxide nanoparticles (anatase crystalline form), alone and in co-presence. Cytological analyses, and histochemical and biochemical evaluation of oxidative stress were carried out on isolated shoots and roots. TEM analysis seemed to indicate the absence of physical/chemical interactions between the two nanomaterials. Seedlings under cotreatment showed the greatest cytotoxic and genotoxic effects and high levels of oxidative stress markers associated with growth inhibition. Even if biochemical data did not evidence significant differences between materials treated with polystyrene nanoplastics alone or in co-presence with titanium dioxide nanoparticles, histochemical analysis highlighted a different pattern of oxidative markers, suggesting a synergistic effect by the two nanomaterials. In accordance, the fluorescence signal linked to nanoplastics in root and shoot was higher under cotreatment, perhaps due to the well-known ability of titanium dioxide nanoparticles to induce root tissue damage, in this way facilitating the uptake and translocation of polystyrene nanoplastics into the plant body. In the antioxidant machinery, peroxidase activity showed a significant increase in treated roots, in particular under cotreatment, probably more associated with stress-induced lignin synthesis than with hydrogen peroxide detoxification. Present results clearly indicate the worsening by metal nanoparticles of the negative effects of nanoplastics on plants, underlining the importance of research considering the impact of cotreatments with different nanomaterials, which may better reflect the complex environmental conditions.

## 1 Introduction

Plastic production has been growing since the 1950s, to the extent that this material has become the main geological marker of the so-called Anthropocene. The high use of plastic for many industrial purposes and in many everyday life products combined with the poor attention to the management of plastic waste, have determined its global diffusion in all natural environments. Polystyrene (PS) is one of the most important polymers in the modern plastic industry, widely used in building materials, electronics, and broadly used in food packaging (Pilevar et al., 2019). In the general alarm about the spread of plastics in the environment, there are particular concerns about PS, considered as potentially more harmful than other plastic polymers as it is composed of relatively hazardous chemicals (Lithner et al., 2011). Once in the environment, plastics, being not biodegradable, undergo ageing and breakage up to micro (less than 5 mm) and nano (less than 100 nm) particles dimension (Barnes et al., 2009; Mattsson et al., 2015). Nanoplastics, which, given their small size, raise particular concerns, can be absorbed by plants through the atmosphere and soil, and once inside they can move to other organs and possibly penetrate cells (Giorgetti et al., 2020; Spanò et al., 2022). By this way they can enter the food chain (Wang et al., 2022), with possible risk for animal and human health. Data in literature report that nanoplastics in themselves can be detrimental for plants in terms of seed germination, seedling growth, induction of oxidative stress, cytotoxicity and genotoxicity (Zhou et al., 2021; Ekner-Grzyb et al., 2022; Spanò et al., 2022). In addition, once into the environment, they can interact with other pollutants, among which metals, that can be absorbed and transported in the different matrices and biological systems. Indeed, this effect of “Trojan horse” exerted by micro/nanoplastics denotes further hazard of particular concern for living organisms, representing critical research gap to be explored. Among the emerging contaminants that may coexist in the same matrices in which plastic materials accumulate, TiO_2_ nanoparticles (nano-TiO_2_) can be taken into account as potential interactors. They are widely spread in the environment, being used in many everyday-life products, mostly for their characteristics of white pigment (Gupta and Tripathi, 2011). Among the different effects on plants reported for these nanoparticles, both positive and negative (Ruffini Castiglione et al., 2011; Song et al., 2013; Ruffini Castiglione et al., 2014; Bellani et al., 2020), their protective action under abiotic stress conditions has also been recorded (Spanò et al., 2019). The interplay between polystyrene nanoplastics (nano-PS) and metal nanoparticles might induce alterations in their uptake by plant, strengthening or mitigating the effects induced if present independently, but, as yet there is very little literature on the subject, especially in relation to plant systems (Das et al., 2022; Guo et al., 2023).

Lentils are widely cultivated high protein legumes, very important for the diet especially in West Asia, East, and North Africa (Alam et al., 2019). They have been used in toxicological studies on the effects of environmental stress factors on growth and development (Zeroual et al., 2023), so much so that they can be considered a model crop in studies on the effects of nanomaterials on plants. For these reasons *Vicia lens* (L.) Coss. & Germ. was chosen in the present experimental design, developed to evaluate if nano-PS and nano-TiO_2_ co-presence could alter their toxicity depending on their potential ability to interact by forming complexes that can exacerbate or mitigate the effects of the two nanomaterials when supplied individually. To achieve this aim, we employed an integrated approach, in short term treatments, conducted in laboratory conditions, encompassing different cytological, physiological, histochemical and biochemical endpoints. For nano-TiO_2_ treatments, we selected a middle-low exposure concentration, 50 mgL^-1^, that could potentially replicate real environmental exposure levels, as estimated by probabilistic material-flow modelling studies (Praetorius et al., 2012). Predicting realistic concentrations of nano-PS in soils is challenging with modelling systems. Therefore, we utilized for them the same concentration and size (30 nm) of nano-TiO_2_ to facilitate comparison of the effects of these two different nanomaterials on *V. lens*. The small size (30 nm) was chosen on the basis of previous studies (Giorgetti et al., 2019, 2020) that had shown the ability of particles of comparable size to be taken up by plants.

The exploitation of fluorophore-conjugated nano-PS allowed us to track them within the different root and shoot compartments of our model plant.

## 2 Materials and methods

### 2.1 Plant material and treatments

Seeds of *Vicia lens* (L.) Coss. & Germ., cv. CDC Robin, bought from the organic farm Floriddia (Pisa, Italy), were surface-sterilized for 3 min in 5% sodium hypochlorite and, after washing, they were germinated in glass Petri dishes (30 seeds x 10 dishes for control and each treatment) at 24 °C ± 1 °C, under light intensity of 300 μmol m^−2^ s^−1^ following a 12 h/12 h day/night cycle with a relative humidity of 70%. The different treatments comprised: distilled water (control, C), a suspension of 50 mgL^-1^ TiO_2_ nanoparticles (nano-TiO_2_), a suspension of 50 mgL^-1^ polystyrene nanoplastics (nano-PS), a suspension of 50 mgL^-1^ TiO_2_ nanoparticles and 50 mgL^-1^ polystyrene nanoplastics (nano-TiO_2_+nano-PS). Commercial powder of nano-TiO_2_ was bought from US Research Nanomaterials Inc. (Houston, USA) as anatase crystal phases (nominal size of 30 nm); nano-PS were purchased from Fisher Scientific S.A.S. as polystyrene green fluorescent particles (nominal size of 30 nm). After five days the length of roots and shoots was recorded, and after measurement seedlings were collected and washed. Roots and shoots were isolated and stored at -80°C or fixed as specified below.

### 2.2 TEM observations

Suspensions of nano-TiO_2_, nano-PS and of their combination, at the selected concentration, were used for TEM observations after an interaction time of 72 h from their preparation. A drop (10 μl) of each suspension was placed on TEM grids covered with formvar, allowed to settle and to dry. The grids were observed under a FEI Tecnai G2 Spirit electron microscope at 100 kV, to evaluate morphology, size, and possible aggregation of isolated and mixed particles.

### 2.3 Cytological analysis

Roots from control and treated seedlings were collected after three days of seed imbibition and fixed overnight in ethanol/glacial acetic acid (3: 1 v/v). Root tips were stained following Feulgen procedure and squashed on microscope slides as described in a previous paper (Giorgetti et al., 2011). For each thesis, 3 slides were analyzed, counting at least 1000 nuclei per slide. The material was examined both under a light microscope and under fluorescence at 560 nm, wavelength specific for pararosaniline (Böhm and Sprenger, 1968) with a Zeiss Axio Observer Z.1, equipped with Zeiss Axiocam MRm. The cytological analysis was done to evaluate the mitotic index, (MI, number of mitoses per 100 nuclei), the frequency of the different mitotic phases and the total cytogenetic anomalies (number of aberrations per 100 nuclei).

### 2.4 Determination of hydrogen peroxide and thiobarbituric acid reactive substances

The determination of hydrogen peroxide content in roots and shoots was determined following the method outlined by Jana and Choudhuri (1982). Plant material was homogenized in 50 mM phosphate buffer, pH 6.5, and centrifugated at 6000*g* for 25 minutes. H_2_O_2_ content was assessed by mixing the supernatant with 0.1% titanium chloride in 20% (v/v) H_2_SO_4_ and measuring the absorbance at 410 nm. The H_2_O_2_ concentration was determined using a standard curve and expressed as μmol g^−1^FW.

For the estimation of lipid peroxidation, TBARS (thiobarbituric acid reactive substances) were measured as in Spanò et al. (2017). The concentration of TBARS was quantified as nmol g^−1^ FW, measuring specific absorbance at 532 nm and subtracting non-specific absorbance at 600 nm. Calculations were performed using an extinction coefficient of 155 mM^−1^ cm^−1^.

### 2.5 Phenols and antioxidant enzymes

Total phenols were measured using Folin-Ciocalteu reagent, according to Arezki et al. (2001). Phenolic extracts obtained after homogenization of plant material in HCl 0.1 N, were added to Folin-Ciocalteu reagent, left so for 3 min in the dark, and incubated at 100°C for 1 min in the presence of Na_2_CO_3_ (20% w/v). After cooling, the absorbance at 750 nm was read. Level of phenolic compounds was expressed as equivalent of gallic acid (GAE mg g^-1^ FW) on the base of a standard calibration curve.

For antioxidant enzymes, extraction was made as in Spanò et al. (2013) after grounding of plant material in liquid nitrogen and homogenization in 100 mM potassium phosphate buffer (pH 7.5). Ascorbate peroxidase (APX, EC 1.11.1.11) activity was measured according to Nakano and Asada (1981) following the decrease in absorbance at 290 nm (extinction coefficient 2.8 mM^−1^ cm^−1^) as ascorbate was oxidized. Catalase (EC 1.11.1.6) activity was determined according to Aebi (1984) and calculated using the 39.4 mM^−1^ cm^−1^ extinction coefficient for hydrogen peroxide. Guaiacol peroxidase (POX, EC 1.11.1.7) activity was determined as described in Arezki et al. (2001) determining guaiacol oxidation by H_2_O_2_ at 470 nm (extinction coefficient 26.6 mM^−1^ cm^−1^), one unit oxidizing 1.0 μmol guaiacol per min. The assessment of all enzymatic activities was conducted at 25°C and expressed as U mg^-1^ protein. Protein quantification was carried out according to the method described by Bradford (1976), using bovine serum albumin (BSA) as a standard.

### 2.6 Electrophoretic peroxidase separation

Electrophoresis was performed on 10% PAGE as in Sorce et al. (2017), using Tris-HCl 1.5 M pH 8.8. After extraction from roots and shoots, equal amounts (15 μg) of proteins were loaded onto electrophoretic gel. After running (200 V, constant current of 35 mA gel^-1^), gels were incubated (in the dark, for 90 min) in 1 M Na-acetate buffer pH 4.6 containing 0.04% benzidine and 10 mM H_2_O_2_. POX activity was visualized as dark brown bands.

### 2.7 Plant anatomy and *in situ* assessment of oxidative stress markers and nanoplastics

Ten roots and shoots, each of similar size and length, sourced from randomly chosen seedlings for each treatment, were isolated, and sliced using a hand microtome. The roots were dissected approximately 3–4 mm from the tip, while the shoots were cut at the beginning of the first internode.

Control samples underwent a general organ anatomical assessment by staining slices with toluidine blue (Bartoli et al., 2017).

Amplex UltraRed Reagent (Life Technologies, USA) was applied to root cross sections for *in situ* detection of H_2_O_2_ (Ruffini Castiglione et al., 2016). After staining, slices were mounted in glycerol and observed with fluorescence microscope (568ex/681em nm). BODIPY 581/591 C11 was applied as a fluorescent marker to visualize lipid peroxidation levels with a change of the fluorescence emission peak from red to green (Spanò et al., 2022). Microscope analysis was performed acquiring simultaneously the green (485ex/510em nm) and the red fluorescence (581ex/591em nm) signals and merging the two images.

To avoid interference with the red autofluorescence of chlorophyll, for the shoot we used probes whose signal was detectable by light microscopy. *In situ* localization of hydrogen peroxide in shoot was performed by 3,3′-diaminobenzidine (DAB) staining (Spanò et al., 2019). This compound is oxidized by hydrogen peroxide occurring in plant cell/tissues to generate dark-brown precipitates. Shoot cross sections were soaked in a freshly prepared incubation medium containing 1 mg mL^−1^ DAB for 4 h at 25°C in complete darkness, then lightened in 96% ethanol at 65°C for 60 min. After extensive rinsing, the samples were immediately observed under light microscopy. Histochemical determination of lipid peroxidation was performed with Schiff’s reagent (Menicagli et al., 2022) (VWR Chemicals BDH) that, binding to free aldehyde groups, can be considered a qualitative indicator of lipid peroxidation. Shoot cross sections were incubated with the dye for 60 min at room temperature, then bleached in 96% ethanol for 60 min at 65°C and analyzed under light microscope to evaluate the developed purple color. Utilizing nano-PS labeled with a green fluorophore enabled us to trace their presence within various root and shoot compartments by examining cross-sections under a fluorescent microscope (468ex/508em nm).

Fluorescence microscope analyses were carried out with a Leica DMLB, equipped with appropriate sets of excitation/emission filters and with a Leica DFC7000 T camera. Optical microscope analyses were performed with a Leitz Diaplan microscope, and images were captured using a Leica DFC 420 camera.

### 2.8 Statistical analysis

The data were expressed as the mean ± standard error (SE), derived from a minimum of four replicates, except when otherwise specified. A significance threshold of *P* < 0.05 was employed. One-way analysis of variance (ANOVA) was carried out, followed by Tukey’s multiple comparison *post-hoc* test.

## 3 Results

### 3.1 Root and shoot anatomy

**Figure 1:**
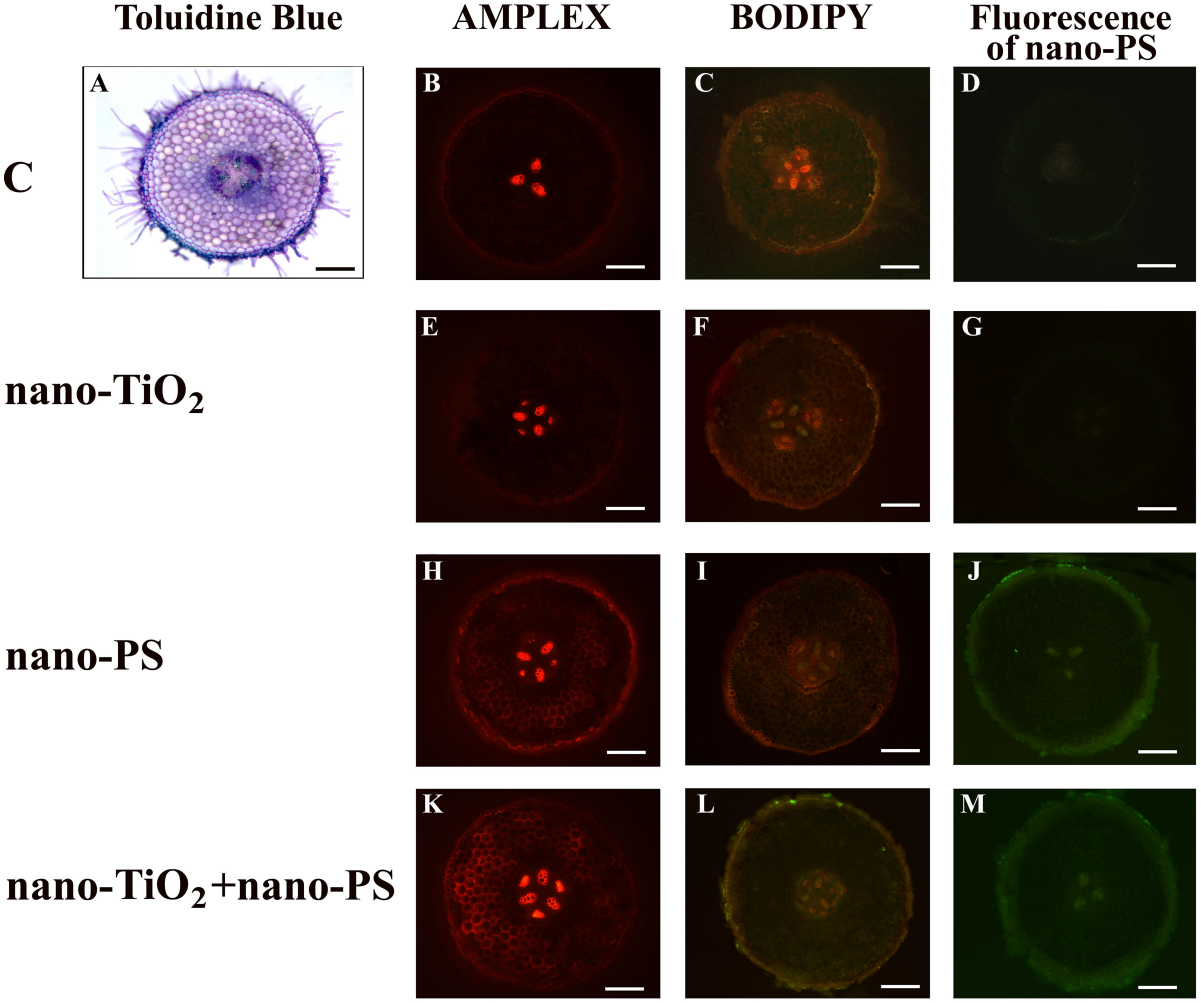
Cross hand sections of *V. lens* roots of seedlings after 5 days of imbibition in water (C) **(A–D)**, in the presence of titanium dioxide nanoparticles (nano-TiO_2_) **(E–G)**, of polystyrene nanoparticles (nano-PS) **(H–J)** and in co-presence of the two nanomaterials (nano-TiO_2_+nano-PS) **(K–M)**. The plate comprehends representative images of root sections stained with Amplex Ultrared probe for *in situ* detection of hydrogen peroxide **(B, E, H, K)**, stained with BODIPY probe for *in situ* detection of TBARS **(C, F, I, L)**, or just analyzed to detect the green fluorescence of nano-PS **(D, G, J, M)**. A representative root cross section of control samples stained with Toluidine Blue is also shown **(A)**. Bars= 200 µm.

To describe the anatomy of lentil, root and shoot sections were analyzed after Toluidine Blue staining. Root cross-sections (**Figure 1A**) were performed in the maturation area, enabling the differentiation of various layers from the outermost to the innermost: rhizodermis with prominent root hairs, exodermis, cortical parenchyma, and endodermis. The central cylinder exhibited organization with a pericycle defining a triarch stele.

**Figure 2:**
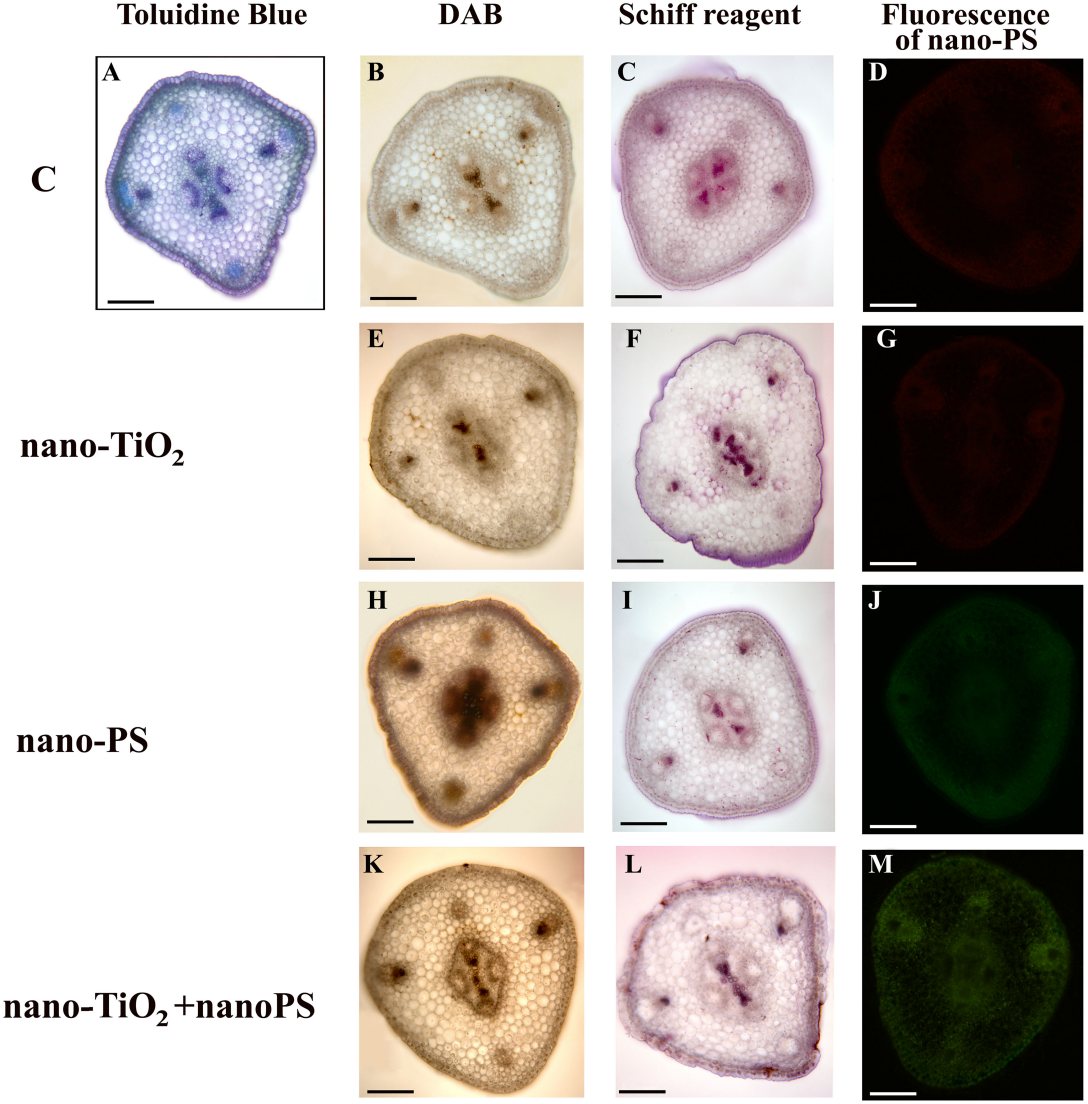
Cross hand sections of *V. lens* shoots of seedlings after 5 days of imbibition in water (C) **(A–D)**, in the presence of titanium dioxide nanoparticles (nano-TiO_2_) **(E–G)**, of polystyrene nanoplastics (nano-PS) **(H–J)** and in co-presence of the two nanomaterials (nano-TiO_2_+nano-PS) **(K–M)**. The plate comprehends representative images of shoot sections stained with DAB probe for *in situ* detection of hydrogen peroxide **(B, E, H, K)**, stained with Schiff reagent probe for *in situ* detection of TBARS **(C, F, I, L)**, or just analyzed to detect the green fluorescence of nano-PS **(D, G, J, M)**. A representative cross section of control samples stained with Toluidine Blue is also shown **(A)**. Bars= 200 µm.

Shoot cross-sections (**Figure 2A**) allowed to distinguish the epidermic layer, cortical sclerenchyma, cortical parenchyma with cortical bundles, typical of species with winged or grooved stem. Vascular stele appeared in a transition structure not yet organized in a true eustele.

### 3.2 Uptake and translocation of nanoplastics

The utilization of nano-PS labelled with fluorophores enabled us to monitor their presence within various compartments of both roots and shoots in our model plant. In root samples treated with nanoplastics, a faint and spread-out fluorescent green signal was observed, intensifying notably in integumental and vascular tissues, especially for cotreated samples (**Figures 1J, M**). Nano-PS were translocated from the root to the shoot, the green fluorescent signal being clearly visible in sections of samples treated with nanoplastics as such or in cotreatment, the latter with greater green color intensity (**Figures 2J, M**). No fluorescent signal was detectable in sections obtained from samples treated with water (C, **Figures 1**, **2D**) or with metal nanoparticles alone (nano-TiO_2_, **Figures 1**, **2G**).

### 3.3 TEM observations

**Figure 3:**
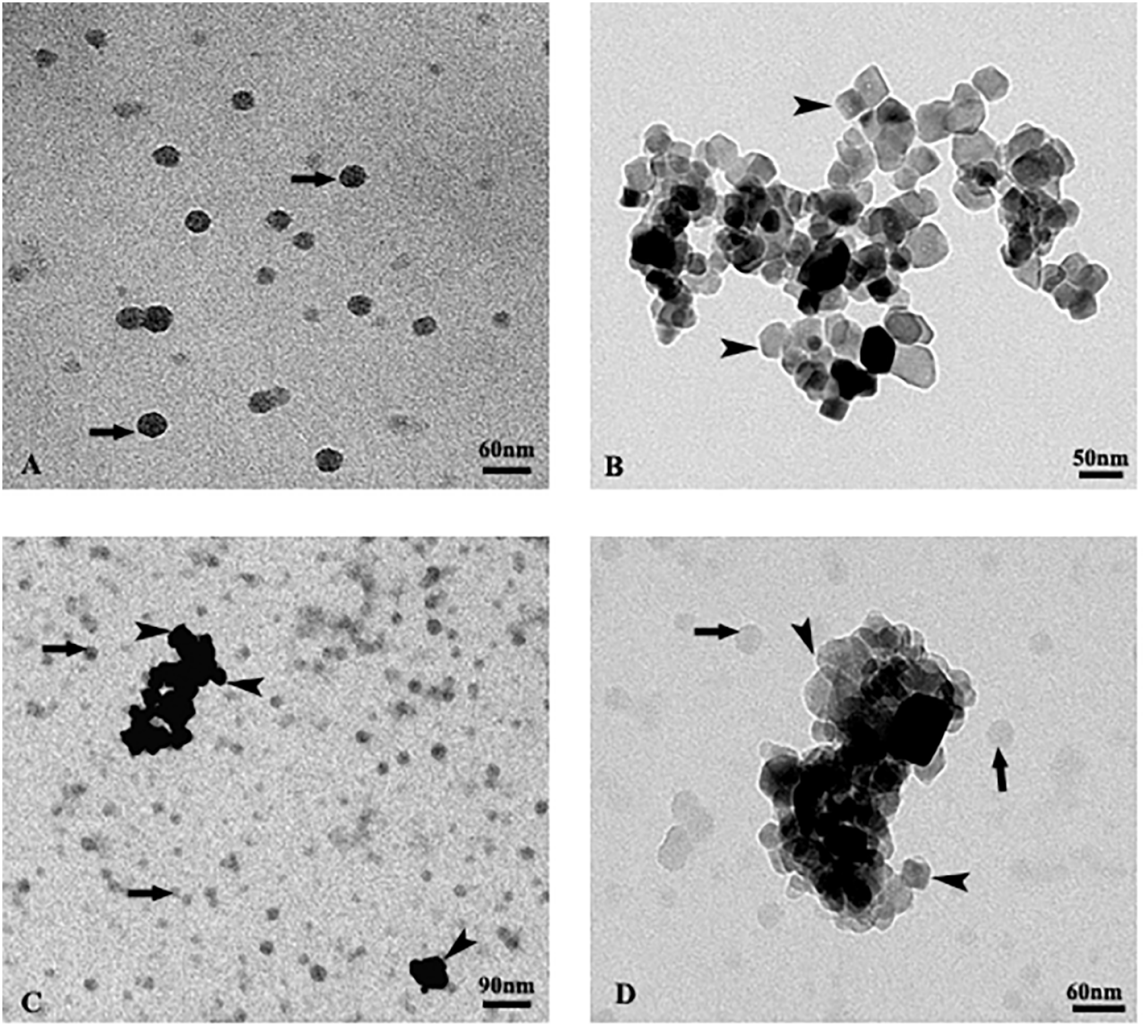
TEM images of isolated polystyrene nanoplastics **(A)**, isolated TiO_2_ nanoparticles **(B)**, mixture of both particles **(C, D)**. The arrows indicate nanoplastics, the arrow heads nanoparticles.

Isolated nano-PS showed regular, almost rounded shape and diameters ranging from 20 to 50 nm. They appeared mostly dispersed, rarely forming aggregates of at most 3 particles (**Figure 3A**).

Isolated nano-TiO_2_ were extremely heterogeneous in shape. They generally appeared prismatic in shape, with size ranging from 20 to 80 nm. They formed large aggregates and rarely were observed isolated (**Figure 3B**).

The suspension containing the mixture of both particles (**Figures 3C, D**) showed no evident interaction between the two types of nanoparticles. In particular, the nano-PS appeared randomly dispersed and not adjacent to the aggregates of the nano-TiO_2_, both keeping the same characteristics as when observed isolated (**Figures 3C, D**).

### 3.4 Seedling growth

The effects of treatments on growth were different in the different organs. Only nano-PS, also in cotreatment with nano-TiO_2_, induced a significant inhibition in root length (**Table 1**), while in shoots (**Table 1**) both nano-TiO_2_ and nano-PS, also in cotreatment, significantly reduced growth. Interestingly, the highest reduction in length was always recorded in seedlings treated with nano-PS.

**Table 1:** Roots length and shoots length of *V. lens* after 5 days of seed imbibition in water (control, C) in the presence of titanium dioxide nanoparticles (nano-TiO_2_), of polystyrene nanoplastics (nano-PS) and in co-presence of the two nanomaterials (nano-TiO_2_+nano-PS).

	Roots length (cm)	Shoots length (cm)
**C**	2.21 ± 0.13 a	2.19 ± 0.09 a
**nano-TiO_2_**	1.86 ± 0.11 ab	1.81 ± 0.09 b
**nano-PS**	1.26 ± 0.06 c	1.68 ± 0.07 b
**nano-TiO_2_+nano-PS**	1.60 ± 0.11 bc	1.78 ± 0.07 b

Within column, values followed by different letters are statistically significant with Tukey test for *P* ≤ 0.05.

### 3.5 Cytological analysis

Root meristems of the primary lentil root were analyzed to determine the possible cytogenetic abnormalities induced by the different treatments taking into account mitotic index and the disturbance of mitotic phases in terms of c-metaphases, abnormal anaphases with chromosomal bridges and fragments and abnormal mitotic spindles.

**Figure 4:**
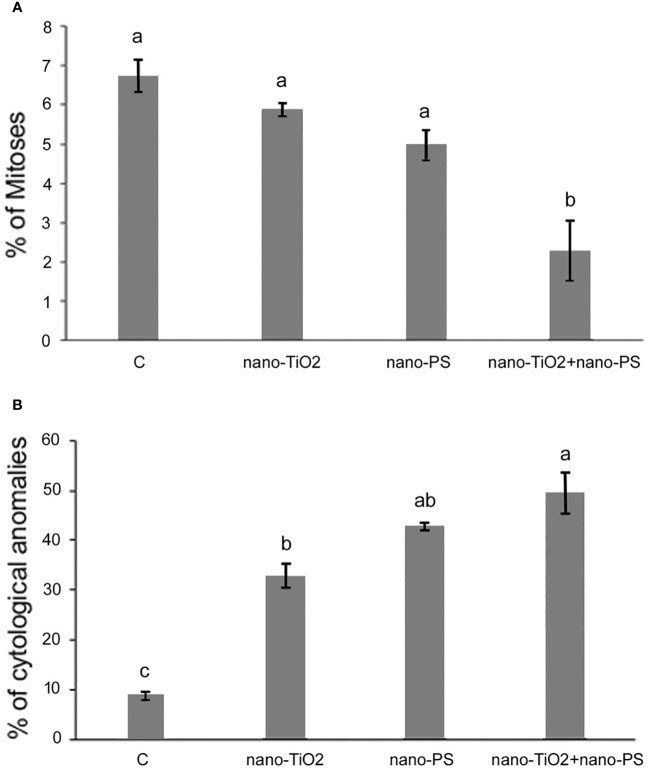
Mitotic index **(A)** and cytological anomalies **(B)** in root meristems of *V. lens* seedlings after 3 days of imbibition in water (C), in the presence of titanium dioxide nanoparticles (nano-TiO2), of polystyrene nanoplastics (nano-PS) and in co-presence of the two nanomaterials (nano-TiO2+nano-PS). Data are expressed as mean values ± standard error. Different letters above bars denote significant differences (*P* ≤ 0.05) among treatments (Tukey test).

The analysis of the mitotic index (MI) showed a decrease of the frequency of cell divisions in treatments with nanoparticles compared to the control (C, MI = 6.73%; nano-TiO_2_, MI = 5.86%; nano-PS, MI = 4.97%, nano-TiO_2_+ nano-PS MI = 2.26%) (**Figure 4A**). The differences were greater and statistically significant in the treatment with the two types of nanoparticles in combination, nano-TiO_2_ + nano-PS, where the reduction was approximately 66.4% compared to the control (**Figure 4A**).

**Figure 5:**
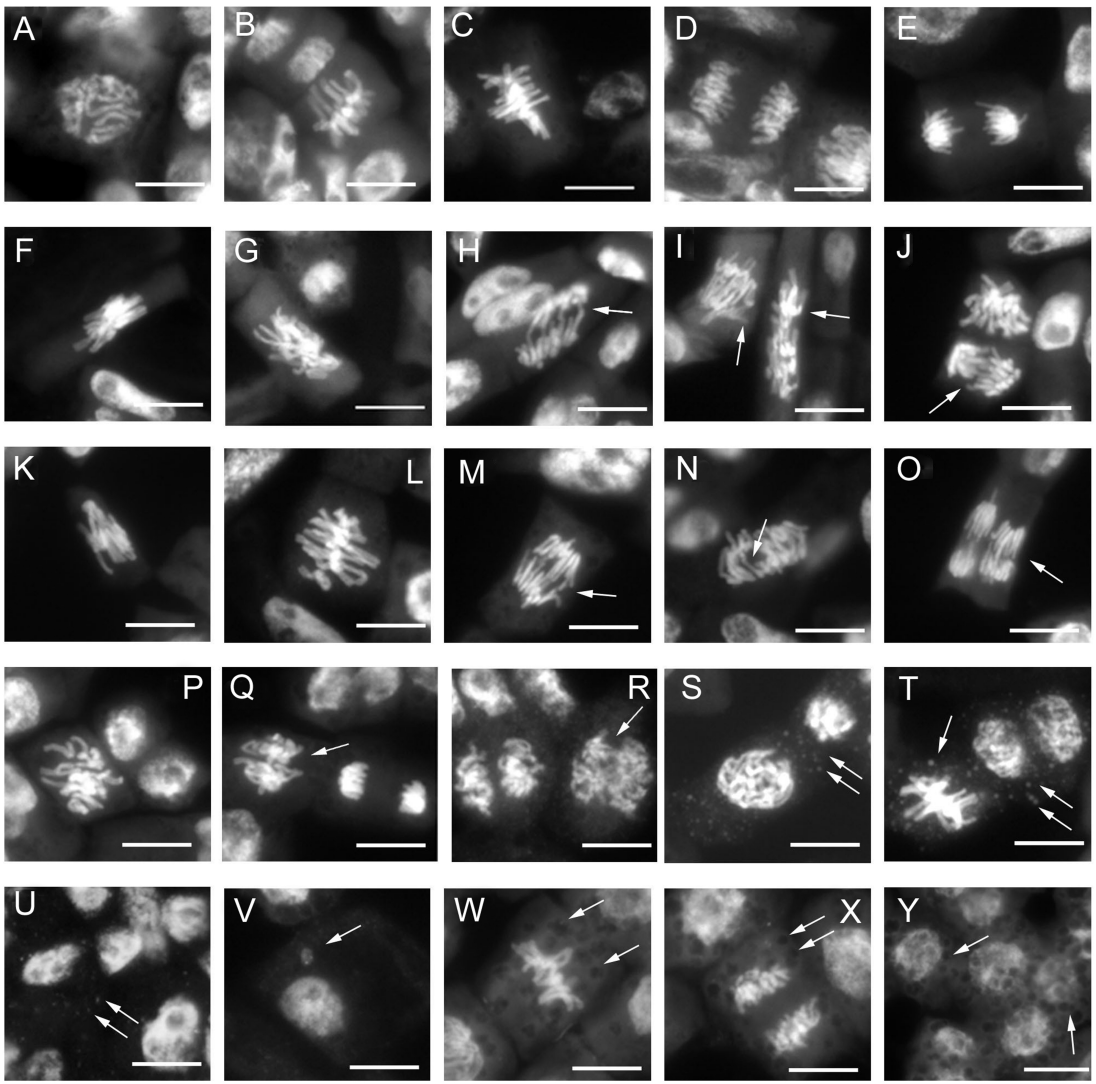
Cytological analysis of 3-day control and treated root meristems of *V. lens*. **(A–E)** control: **(A)** normal prophase; **(B, C)** normal metaphase;**(D, E)** normal anaphase; **(F-J)** nano-TiO_2_ treatment: **(F)** sticky chromosomes in metaphase; **(G)** c-metaphase; **(H–J)** abnormal anaphases with chromosome bridges **(H)** and faulty spindles **(I, J)**; **(K–O)** nano-PS treatment: **(K, L)** sticky chromosomes in metaphase, **(M–O)** chromosomes bridges at anaphases; **(P–Y)** nano-TiO_2_+ nano-PS treatment: **(P)** c- metaphase, **(Q–R)** abnormal anaphases with defective mitotic spindles, **(S–U)** Feulgen positive bodies (arrows) in the cytoplasm of meristematic cell in prophase **(S)**, metaphase, ana-telophase **(T)** and in interphase cells, **(V)** micronucleus; **(W–Y)** evident cytoplasmic vesicles (arrows) observed in cytoplasm of metaphase **(W)** anaphase **(X)** and interphase cells. Arrows indicate cytogenetic anomalies and cytoplasmic vesicles. Bars = 10 µm.

The analysis of mitotic phases frequencies is shown in **Table 2**. No significant effects were found concerning the frequency of prophase, but abnormal metaphases and anaphases significantly increased in treated root meristems with the maximum reached for nano-TiO_2_ + nano-PS (abnormal metaphases 28.29%, abnormal anaphases 20.46%). The increase in the total cytogenetic abnormalities was clearly shown in **Figure 4B**. In particular, the cotreatment with nano-TiO_2_ and nano-PS induced the greatest number of anomalies (49.45%), much higher than the treatment with nano-TiO_2_ (32.8%) and nano-PS (42.96%) alone. Some representative examples of cytological anomalies are shown in **Figure 5**. Unlike the control, in which mainly normal mitoses were observed (**Figures 5A-E**), in all treatments with nanoparticles it was possible to observe sticky chromosomes (**Figures 5F, K, L**), c-metaphases (colchicine-like metaphases, caused by partial or complete spindle failure at mitosis) (**Figures 5G, P**), abnormal anaphases with bridges (**Figures 5H-J**), lagging chromosomes (**Figures 5M-O**) and abnormal mitotic spindles (**Figures 5I-J, Q, R**). Moreover, in nano-TiO_2_ + nano-PS treatment, other cytological abnormalities were observed in the cytoplasm of both mitotic and interphase cells, such as the presence of Feulgen-positive bodies (**Figures 5S, T, U**), the presence of micronuclei (**Figure 5V**) and cytoplasmic vesicles (**Figures 5W, X, Y**).

**Table 2:** Cytological analysis in root meristems of *V. lens* after 3 days of seed imbibition in water (control, C) in the presence of titanium dioxide nanoparticles (nano-TiO_2_), of polystyrene nanoplastics (nano-PS) and in co-presence of the two nanomaterials (nano-TiO_2_+nano-PS).

	C	nano-TiO_2_	nano-PS	nano-TiO_2_+nano-PS
**% Normal Prophases**	19.90 ± 1.76 a	15.24 ± 2.16 a	20.45 ± 2.61 a	21.29 ± 1.43 a
**% Normal Metaphases**	29.68 ± 4.58 b	22.79 ± 1.8 b	13.34 ± 1.05 a	13.89 ± 3.48 a
**% Abnormal Metaphases**	4.05 ± 1.16 a	18.59 ± 3.09 a	24.18 ± 1.49 a	28.29 ± 8.5 b
**% Normal Anaphase/telophases**	41.53 ± 4.45 b	29.16 ± 3.29 ab	23.24 ± 2.69 a	15.37± 4.64 a
**% Abnormal Anaphase/Telophases**	4.84 ± 0.98 a	14.22 ± 1.16 a	18.79 ± 0.79 b	20.46 ± 7.36 b

Values are expressed as a means of percentage analyzed in 3 replicates (n=3 ± ES). Within row, values followed by different letters are statistically significant with Tukey test for *P* ≤ 0.05.

### 3.6 Biochemical and histochemical analyses of oxidative stress

**Figure 6:**
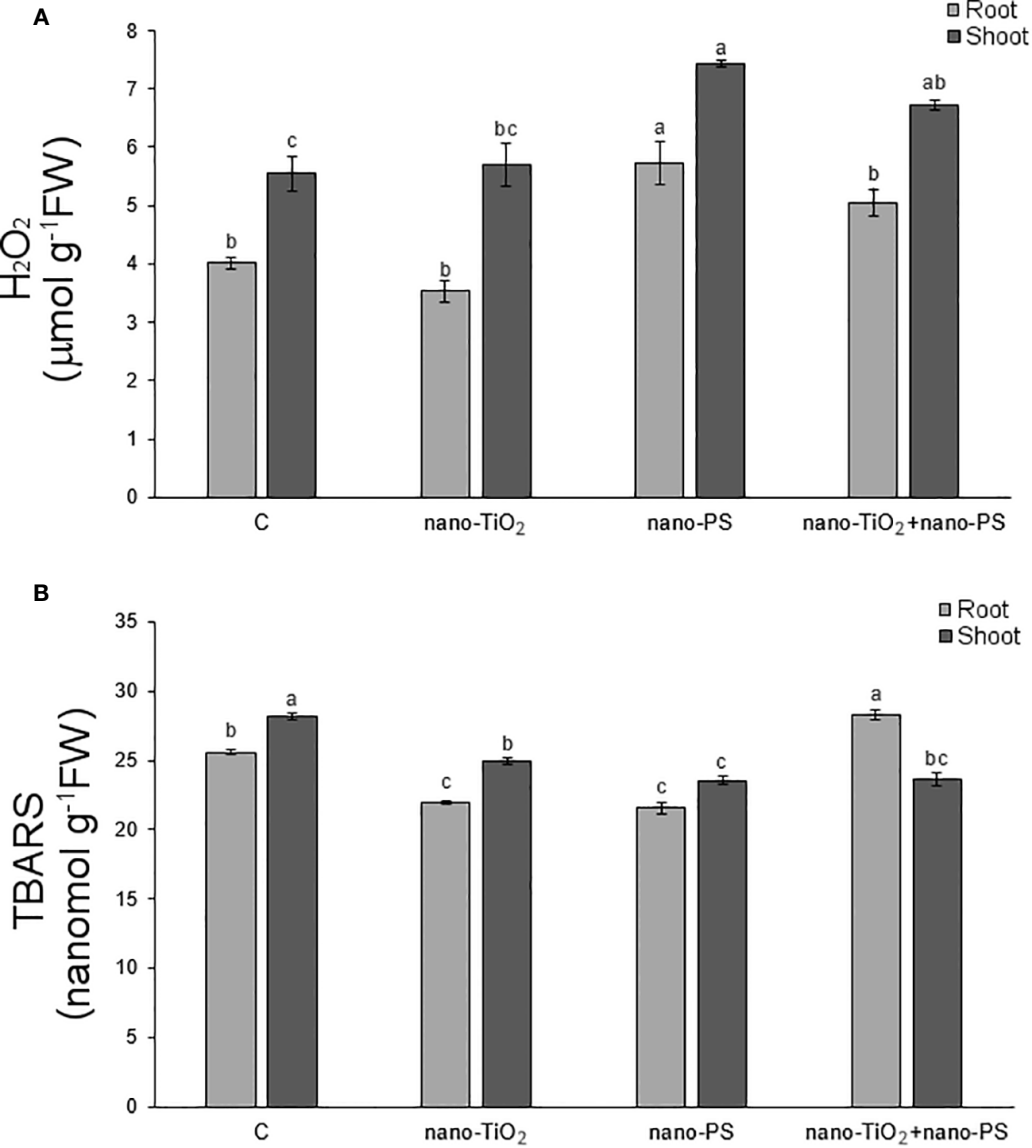
Hydrogen peroxide **(A)** and thiobarbituric acid reactive substances (TBARS, **B**) in roots and shoots of *V. lens* seedlings after 5 days of imbibition in water (C), in the presence of titanium dioxide nanoparticles (nano-TiO_2_), of polystyrene nanoplastics (nano-PS) and in co-presence of the two nanomaterials (nano-TiO_2_+nano-PS). For each organ, different letters above bars denote significant differences (*P* ≤ 0.05) among treatments (Tukey test).

In our experimental conditions, the concentration of hydrogen peroxide (**Figure 6A**) was significantly higher in shoots and roots of seedlings treated with nano-PS, both alone and in cotreatment with nano-TiO_2_. The highest values were always detected under nano-PS treatment. Oxidative damage, estimated as TBARS (**Figure 6B**), was generally lower in treated seedlings in comparison with controls. Only in roots cotreatment induced the highest damage, significantly higher than that recorded in all other treatments. Histochemical probes, proposed specifically for hydrogen peroxide and lipid peroxidation, as illustrated in **Figures 1** and **2**, allowed to identify qualitative signals indicative of oxidative stress. In the cross sections of both control and treated roots, the red fluorescent probe Amplex, specific for H_2_O_2_, yielded a signal mostly localized within xylem vessels, with varying intensity staining observed in the root epidermis (**Figure 1**). In nano TiO_2_-treated samples, the red signal extended to include phloem arches (**Figure 1E**), while nanoplastic-treated samples exhibited staining, particularly noticeable in the tegumental tissues and partially covering the cortex (**Figure 1H**). Cotreatment resulted in a heightened response within the vascular cylinder, extending nearly across the entire cortical cylinder, being the most responsive treatment to Amplex staining (**Figure 1K**). Cotreated sample exhibited the highest reactivity also in relation to the BODIPY fluorescent probe, as depicted in **Figure 1L**. This probe identifies lipid peroxidation by detecting a shift in fluorescence emission from red to green. In the cotreated sample, green fluorescence was consistently observed in all root tissues, encompassing both cortical and vascular cylinder regions (**Figure 1L**). In the other samples, the signal was weakly diffused over the whole section (**Figures 1F, I**), with the control showing specifically a general signal absence in the vascular tissues (**Figure 1C**).

The histochemical assessment of hydrogen peroxide (H_2_O_2_) using DAB in shoot cross sections of control samples indicated positive signals, as brown precipitates, concentrated in the xylem of both cortical bundles and stellar vascular bundles (**Figure 2B**). Treated samples exhibited a similar staining pattern, albeit more diffuse within the shoot (**Figures 2E, H, K**), with the enhancement being particularly notable in plants treated with nano-PS (**Figure 2H**).

The *in situ* detection of lipid peroxidation using Schiff’s reagent, as a magenta color development, did not reveal distinct differences in the staining pattern (**Figures 2F, I, L**), except for the tegmental tissues that demonstrated heightened responsiveness in samples treated with nano-TiO_2_, either alone or in cotreatment with nano-PS (**Figures 2F, L**).

### 3.7 Antioxidant response

**Figure 7:**
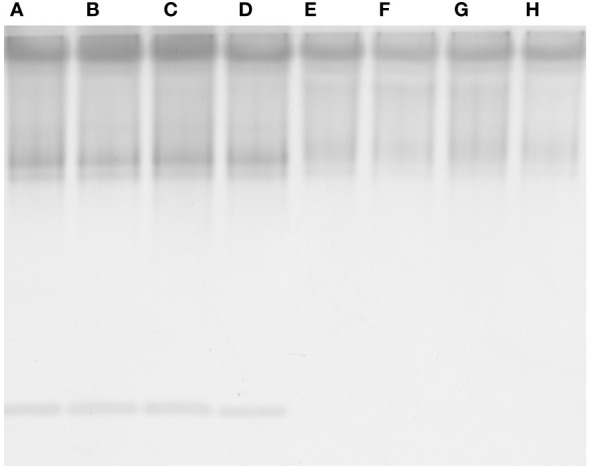
Native polyacrylamide gel electrophoresis of guaiacol peroxidase from roots **(A-D)** and shoots **(E-H)** of *V. lens* seedlings after 5 days of imbibition in water (control, **A**, **E**) in the presence of titanium dioxide nanoparticles **(B, F)**, of polystyrene nanoplastics **(C, G)** and in co-presence of the two nanomaterials **(D, H)**.

The highest concentration of phenols was measured in seedlings treated with nano-PS, though only in shoot the value was significantly higher than in controls (**Tables 3**, **4**). In root, APX and CAT activities were lower in all treatments than in control (**Table 3**). Nano-PS-treated plants were characterized by the lowest catalase activity. An opposite trend was recorded for POX activity, that was higher in treated plants in comparison to control, though the differences were significant under nano-PS alone or in cotreatment with nano-TiO_2_. In shoot there were no differences among control and different treatments, with the only exception of CAT activity, that was significantly higher in nano-TiO_2_-treated plants than in all other materials (**Table 4**). After electrophoresis (**Figure 7**), specific staining for revealing POX activity highlighted bands with different mobility, with distinct patterns depending on the organ, but not on the different treatment. Noteworthy is the presence of a band with greater mobility detectable only in the roots, in accordance with the higher activities recorded in this organ in comparison with shoots.

**Table 3:** Phenols content (equivalent of gallic acid, GAE mg g^-1^ FW), and ascorbate peroxidase, guaiacol peroxidase, catalase activities (U mg^-1^ protein) in roots of *V. lens* after 5 days of seed imbibition in water (control, C) in the presence of titanium dioxide nanoparticles (nano-TiO_2_), of polystyrene nanoplastics (nano-PS) and in co-presence of the two nanomaterials (nano-TiO_2_+nano-PS).

	Phenols	APX	POX	CAT
**C**	0.77 ± 0.02 a	0.68 ± 0.01 a	4.36 ± 0.06 b	5.05 ± 0.31 a
**nano-TiO_2_**	0.59 ± 0.00 b	0.62 ± 0.01 b	5.05 ± 0.19 ab	2.61 ± 0.22 b
**nano-PS**	0.78 ± 0.00 a	0.61 ± 0.02 b	5.56 ± 0.49 a	1.72 ± 0.06 c
**nano-TiO_2_+nano-PS**	0.57 ± 0.03 b	0.61 ± 0.01 b	5.85 ± 0.14 a	2.31 ± 0.17 bc

Within column, values followed by different letters are statistically significant with Tukey test for *P* ≤ 0.05.

**Table 4:** Phenols content (equivalent of gallic acid, GAE mg g^-1^ FW), and ascorbate peroxidase, guaiacol peroxidase, catalase activities (U mg^-1^ protein) in shoots of *V. lens* after 5 days of seed imbibition in water (control, C) in the presence of titanium dioxide nanoparticles (nano-TiO_2_), of polystyrene nanoplastics (nano-PS) and in co-presence of the two nanomaterials (nano-TiO_2_+nano-PS).

	Phenols	APX	POX	CAT
**C**	0.82 ± 0.02 b	0.61 ± 0.05 a	2.88 ± 0.11 a	2.89 ± 0.21 b
**nano-TiO_2_**	0.72 ± 0.02 c	0.55 ± 0.01 a	2.46 ± 0.23 a	5.06 ± 0.27 a
**nano-PS**	0.95 ± 0.03 a	0.57 ± 0.04 a	2.32 ± 0.28 a	2.83 ± 0.23 b
**nano-TiO_2_+nano-PS**	0.71 ± 0.00 c	0.56 ± 0.01 a	2.72 ± 0.26 a	3.08 ± 0.09 b

Within column, values followed by different letters are statistically significant with Tukey test for *P* ≤ 0.05.

## 4 Discussion

The widespread dispersal of plastic debris in all the ecosystems results in the emergence of deemed more hazardous contaminants like micro and nanoplastics. In real-life situations, organisms frequently encounter various microplastics/nanoplastics and other pollutants, including metal nanoparticles. They also represent a class of emerging contaminants of particular concerns, which production and environmental release is increasing due to their large use in different applications and in nanotechnologies.

In fact, it is considered likely that plastics interact with other pollutants (Mohana et al., 2022), and when combined, these interactions could either amplify or mitigate their individual phytotoxic effects. Nonetheless, there is a limited body of research on the interplay between nanoplastics and metal nanoparticles and on the underlying mechanisms of their action on plants. In contrast to data in the literature (Das et al., 2022; Natarajan et al., 2022) our results seem to indicate the absence of interactions between the two nanomaterials used. These differences could, however, be explained if the different nature of the experimental system and of the nanomaterials are considered. First of all, the crystalline form of titanium used in the present experiment was anatase, while a mixture of anatase and rutile was employed in the previous works. Secondly, nanoplastics are quite different from those used previously, with a smaller size, comparable to that of nano-TiO_2_. The lack of surface functional groups, in addition, could have an impact on their ability to absorb contaminants (Wang et al., 2021).

An examination of the detrimental impacts of environmental pollutants on living organisms should encompass an analysis of their possible cyto-genotoxic effects. The investigation of root meristem behavior is a valuable approach in this regard. Although lentil (2n 14) is not a model species for genotoxicity studies, cytological analysis has evidenced the most common cytogenetic anomalies found in treated meristems, which consist of c-metaphases, sticky metaphases, anaphases with bridges and abnormal mitotic spindles. As previously reported, both TiO_2_ nanoparticles (Ruffini Castiglione et al., 2016) and nanoplastics (Muccifora et al., 2022; Spanò et al., 2022) can induce genotoxicity in plant systems. In the present experiment the greatest cytotoxic (lowering of mitotic activity) and genotoxic effect (increase of chromosomal abnormalities) was observed in the cotreatment with nano-TiO_2_+nano-PS, giving evidence of the negative synergistic effect of the two types of nanoparticles. This result agreed with what has been reported in the *Scenedesmus obliquus* algae (Das et al., 2022) in which it was observed that nano-PS played a pivotal role in enhancing the toxicity of nano-TiO_2_ in freshwater algae. Genotoxicity can be determined by a direct action of nanoparticles at DNA and mitotic spindle level and/or by oxidative stress and ROS production. Given that oxidative stress is at the basis of the negative effects induced by nanomaterials, it has been suggested as the main driver of the response of higher plants to such exposures (Gao et al., 2023), starting from the early stages of development. Even if these treatments applied do not lead to an alteration of the general anatomy of the seedling, disturbances in growth were recorded. Both negative and positive effects of nano-PS on seedling growth are reported in literature (Lian et al., 2020; Spanò et al., 2022). In the present experiment, the decrease in the length of roots and shoots induced by nano-PS was associated with the highest concentration of hydrogen peroxide, marker of oxidative stress. The nano-TiO_2_ themselves had a negative impact only on shoot growth, with no significant action on root length. This is further confirmation of data present in the literature (Li et al., 2023) which underlines that nanoparticles can induce different responses depending on the organ considered. The addition of nano-TiO_2_ to nano-PS was neither able to alleviate the negative effect of plastics on the root growth nor to reduce the concentration of the H_2_O_2_. As widely accepted, an overproduction of ROS, can induce oxidative damage to cellular structures and macromolecules. To avoid injury, plants have evolved both enzymatic and non-enzymatic antioxidant machinery. Enzymes like APX, CAT, and POX play a crucial role in the breakdown of H_2_O_2_. From a biochemical point of view, oxidative damage, estimated as TBARS, was always less than the control. A similar decrease, under nanomaterial treatment, has been previously documented in *A. cepa* (Giorgetti et al., 2020), and could be due to an effective antioxidant response. In fact, in our system, increased POX activity in roots for nano-TiO_2_ and nano-PS treatments, and elevated phenol content and CAT activity in shoots following nano-PS and nano-TiO_2_ treatment, respectively (**Tables 3**, **4**) were recorded.

Roots of seedlings under cotreatment were characterized by the highest oxidative injury, also shown in histochemical results, despite the POX activity reached the maximum value. It should be noted that the activity of peroxidases, higher than the control in all treatments, is not only associated with the regulation of hydrogen peroxide levels, but also plays a significant role in the lignification process (Lee et al., 2007), lignin deposition playing an important role in plant adaptation under adverse environmental conditions (Yadav and Chattopadhyay, 2023).

At this point it should be underlined that in the present work, in addition to the biochemical quantitative analysis, *in situ* analysis of relevant toxicological markers has been performed. This is a key approach to uncover localized stress signals in specific root/shoot compartments, indicative of injury for lipid peroxidation but also of developmental and/or defense responses for hydrogen peroxide, which are characteristic of distinct treatments themselves. In root, treatment with nano-TiO_2_ indicated a presence of H_2_O_2_ in the phloem arches (**Figure 1E**), absent in control samples (**Figure 1B**), as also found in *Vicia faba* exposed to nano-TiO_2_ of different size and shape (Ruffini Castiglione et al., 2016) and in *Pisum sativum* under anatase, alone or in a mix with rutile, nano-TiO_2_ being spiked in agricultural soil growth medium (Giorgetti et al., 2019). Root response as peculiar localization of H_2_O_2_ could be linked to an alternative plant stress-related reaction triggered by this nanomaterial and common to different legume species. Nano-PS treatment seems to induce a more extensive pattern of H_2_O_2_, according to biochemical data and resembling the results obtained in *Allium cepa* (Giorgetti et al., 2020) and *Oryza sativa* (Spanò et al., 2022) when treated with nano-PS at higher concentration (1 g L^-1^). This pattern is even more pronounced in cotreatment experimentation, demonstrating synergistic effect by the two nanomaterials. This synergistic effect is likewise evident if the histochemical data relating to lipid peroxidation are observed. The *in situ* analyses of the oxidative stress markers were less conclusive in the shoot, in which no co-acting effects seems to arise. When analyzing the localization of the nano-PS in the two organs, in both root and shoot, the patterning of green fluorescence at the specific excitation wavelengths indicates uptake and translocation of nano-PS (**Figures 1J, M**, **2J, M**), confirming the data obtained in *Cicoria endivia* and *Oryza sativa* seedlings treated with 50 and 20 nm nano-PS respectively (Muccifora et al., 2022; Spanò et al., 2022). Our findings diverged from earlier observations in *Arabidopsis thaliana*, where fluorescent nano and microplastics were not able to overcome plant cell barriers and were detectable on the root surface and calyptra cells (Taylor et al., 2020). The different experimental conditions and the different types of nano/micro plastic materials may be behind the different results obtained, as well as the different plant systems used.

In our experimental system, a higher intensity of fluorescence from nano-plastics was detectable in the cotreated materials both in root and in shoot (**Figures 1M**, **2M**). This suggests that treatment with nano-TiO_2_ would induce root tissue damage by facilitating the entry and uptake of nano-PS into the plant body. In fact, in a previous work (Muccifora et al., 2021), it has been shown that anatases nano-TiO_2_ are able to produce crossing ruptures of cell wall and plasmalemma of adjacent cell root, in this way enhancing nanoplastic uptake.

## 5 Conclusions

Both nano-PS and nano-TiO_2_ were able to induce negative effects on lentil at different levels of organization, from the cell to the whole plant. Overall, stress parameters were higher under cotreatment, in which the uptake of nanoplastics, tracked thanks to their fluorescence, seemed relatively higher than when administered as such. Unlike some cases where nanoplastics can alleviate adverse impacts of other pollutants by aggregating with them, nano-PS, in this study, seems to lack this capability when in cotreatment with nano-TiO_2_, confirming no apparent interaction between them. The absence of interaction between nano-PS and nano-TiO_2_ allows the latter to directly harm cell walls and membranes. This intensifies the absorption of nanoplastics and exacerbates stress symptoms in plants. The data emphasizes the potential danger of the simultaneous presence of these emerging pollutants in the environment, underscoring the need for attention and further investigation into their combined effects.

## Data availability statement

The original contributions presented in the study are included in the article/supplementary material. Further inquiries can be directed to the corresponding author.

## Author contributions

CS: Conceptualization, Data curation, Formal analysis, Investigation, Methodology, Resources, Supervision, Visualization, Writing – original draft, Writing – review & editing. LG: Data curation, Formal analysis, Investigation, Methodology, Resources, Visualization, Writing – review & editing. SB: Data curation, Formal analysis, Investigation, Methodology, Visualization, Writing – review & editing, Conceptualization, Software, Writing – original draft. SM: Data curation, Formal analysis, Investigation, Methodology, Visualization, Writing – review & editing, Resources. MR: Data curation, Formal analysis, Investigation, Methodology, Resources, Visualization, Writing – review & editing, Conceptualization, Supervision, Writing – original draft.
